# Altered T‐cell subset distribution in the viral reservoir in HIV‐1‐infected individuals with extremely low proviral DNA (LoViReTs)

**DOI:** 10.1111/joim.13484

**Published:** 2022-03-28

**Authors:** Cristina Gálvez, Víctor Urrea, Maria del Carmen Garcia‐Guerrero, Sílvia Bernal, Susana Benet, Beatriz Mothe, Lucía Bailón, Judith Dalmau, Andrea Martinez, Aroa Nieto, Lorna Leal, Felipe García, Bonaventura Clotet, Javier Martinez‐Picado, Maria Salgado

**Affiliations:** ^1^ IrsiCaixa AIDS Research Institute and Institute for Health Science Research Germans Trias i Pujol (IGTP) Hospital Germans Trias i Pujol Badalona Spain; ^2^ Chair in Infectious Diseases and Immunity University of Vic – Central University of Catalonia (UVic‐UCC) Vic Spain; ^3^ Lluita contra la SIDA Foundation Infectious Diseases Department Hospital Germans Trias i Pujol Badalona Spain; ^4^ CIBER de Enfermedades Infecciosas Madrid Spain; ^5^ Department of Medicine Autonomous University of Barcelona Catalonia Spain; ^6^ Infectious Diseases Department Hospital Clínic University of Barcelona Barcelona Spain; ^7^ Catalan Institution for Research and Advanced Studies (ICREA) Barcelona Spain

**Keywords:** CD4^+^ T‐cell subpopulations, HIV latency, HIV reservoir, secondary lymphoid tissues, total HIV‐DNA

## Abstract

**Background:**

HIV cure strategies aim to eliminate viral reservoirs that persist despite successful antiretroviral therapy (ART). We have previously described that 9% of HIV‐infected individuals who receive ART harbor low levels of provirus (LoViReTs).

**Methods:**

We selected 22 LoViReTs matched with 22 controls ART suppressed for more than 3 years with fewer than 100 and more than 100 HIV‐DNA copies/10^6^ CD4^+^ T cells, respectively. We measured HIV reservoirs in blood and host genetic factors. Fourteen LoViReTs underwent leukapheresis to analyze replication‐competent virus, and HIV‐DNA in CD4^+^ T‐cell subpopulations. Additionally, we measured HIV‐DNA in rectum and/or lymph node biopsies from nine of them.

**Results:**

We found that LoViReTs harbored not only lower levels of total HIV‐DNA, but also significantly lower intact HIV‐DNA, cell‐associated HIV‐RNA, and ultrasensitive viral load than controls. The proportion of intact versus total proviruses was similar in both groups. We found no differences in the percentage of host factors. In peripheral blood, 71% of LoViReTs had undetectable replication‐competent virus. Minimum levels of total HIV‐DNA were found in rectal and lymph node biopsies compared with HIV‐infected individuals receiving ART. The main contributors to the reservoir were short‐lived transitional memory and effector memory T cells (47% and 29%, respectively), indicating an altered distribution of the HIV reservoir in the peripheral T‐cell subpopulations of LoViReTs.

**Conclusion:**

In conclusion, LoViReTs are characterized by low levels of viral reservoir in peripheral blood and secondary lymphoid tissues, which might be explained by an altered distribution of the proviral HIV‐DNA towards more short‐lived memory T cells. LoViReTs can be considered exceptional candidates for future interventions aimed at curing HIV.

## Graphical abstract

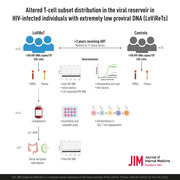

## Introduction

Antiretroviral therapy (ART) effectively blocks viral replication but does not completely eliminate HIV infection [1] owing to the persistence of integrated proviruses, mainly in CD4^+^ T cells. These proviruses constitute the viral reservoir, which is the main barrier to curing HIV. Thus, most HIV‐infected individuals experience a rapid relapse of viremia when ART is interrupted. However, an extremely small proportion of HIV‐infected individuals control viral replication naturally in the absence of ART and usually harbor low viral reservoirs. These individuals are considered examples of functional cure and comprise phenotypes such as elite controllers [2, 3], exceptional elite controllers [4, 5], and post‐treatment controllers [6], in whom cumulative viral and host protective factors are observed [7, 8]. In exceptional elite controllers (and elite controllers), we and others have observed enriched protective HLA alleles, potent cytotoxic CD8^+^ T‐cell activity, and a lack of viral evolution, probably owing to the presence of defective proviruses [4, 9, 10, 11]. In contrast, post‐treatment control is associated with early initiation of ART, enriched HLA‐B*35/07 alleles, weak HIV‐specific cytotoxic activity, and unusual distribution of HIV latency in CD4^+^ T‐cell subpopulations [6].

These exceptional cases have improved our understanding of the mechanisms required to control the virus and that could be emulated through medical interventions in people with HIV who currently require lifelong therapy. Therefore, various strategies are being studied with the aim of reducing the latent HIV reservoir as a first step towards curing HIV [12]. Interestingly, in a previous study by our institution, a group of treated HIV‐infected individuals achieved this aim naturally, harboring low or even undetectable levels of HIV‐DNA under ART. This cohort was named LoViReT (Low Viral Reservoir Treated) [13], and the individuals included account for 9% of the treated HIV‐infected population. We observed that these individuals initiated ART mainly during the chronic phase of infection, in contrast to post‐treatment controllers, which are more frequently found in early treated individuals [14]. This new phenotype is characterized mainly by a naturally reduced viral reservoir and a less impaired CD8^+^ T‐cell compartment before the start of treatment, with a faster and more marked reduction of the reservoir at initiation of ART and in circulating HIV‐specific antibodies after ART [13].

The LoViReT cohort constitutes an optimal platform for HIV cure studies focused on both research into new mechanisms of reservoir reduction and on the study of immune‐based strategies that can facilitate complete viral remission in the context of previous reduction of the HIV reservoir. The latter approach is especially important, since previous studies in participants with a phenotype similar to that of the LoViReT cohort showed that when treatment was stopped, most individuals experienced viral rebound [15, 16]. This observation raises the questions of whether the low reservoirs observed in blood correlate with similar levels of provirus in anatomical compartments and whether the distribution of the reservoir among different T‐cell subpopulations could alter the dynamics of viral persistence.

In this study, we comprehensively analyzed 22 individuals from the LoViReT cohort who had initiated ART during the early and the chronic phases of HIV‐1 infection in order to decipher the nature and mechanism of their low latent HIV reservoir levels. We assessed protective host factors and the distribution and functionality of their proviruses in blood and secondary lymphoid tissues. Understanding the mechanisms by which LoViReT individuals have a reduced latent reservoir will be key to designing a cure strategy for most HIV‐infected persons.

## Methods

### Participants and study design

Of the 42 LoViReT individuals defined in an earlier cohort [13], we selected 22 (both early and chronic treated) who met the inclusion criteria and were willing to participate in the study. We used the random forest method to match them with 22 controls for similarity or proximity with respect to 17 clinical characteristics (e.g., sex, age, CD4^+^ T cells at sampling, and zenith viral load [Fig. S1]). Participants were receiving care at Hospital Germans Trias i Pujol and Hospital Clinic, Barcelona (Spain). The inclusion criteria for both groups were to be receiving suppressive ART with undetectable viremia (HIV‐RNA <50 copies/ml) for at least 3 years, with fewer than 100 HIV‐DNA copies/10^6^ CD4^+^ T cells in the case of LoViReT individuals and more than 100 HIV‐DNA copies/10^6^ CD4^+^ T cells in the case of controls. Participants were classified as early or chronic treated if there were diagnostic criteria to document whether ART had been initiated within 6 months since the estimated acquisition of HIV, as previously defined in the IrsiCaixa Early‐ART Cohort [17].

All the LoViReT individuals were invited to participate in the second phase of the study with a more in‐depth analysis of peripheral blood and secondary lymphoid tissues. A subgroup of 14 individuals agreed to undergo leukapheresis to increase the number of cells needed to perform a quantitative viral outgrowth assay (qVOA) and measure the distribution of virus among CD4^+^ T‐cell subpopulations. Rectum and lymph node biopsies were taken only from those with an infectious units per million (IUPM) value smaller than 0.1 who agreed to participate (Fig. S1). Control individuals did not undergo this second phase of the study.

All participants provided their signed informed consent. The study was approved by the ethics committees at both recruiting centers (reference #: PI‐17‐043).

### Quantification of the HIV reservoir in blood

To evaluate the size of the viral reservoir, lysed extracts from purified CD4^+^ T cells were used to measure total HIV‐DNA by droplet digital polymerase chain reaction (ddPCR), as previously described [18]. Briefly, 5′LTR or *gag* regions were amplified, and the *RPP30* housekeeping gene was measured in parallel to normalize sample input. Raw ddPCR data were analyzed using the QX100™ Droplet Reader and the software application QuantaSoft v.1.6 (Bio‐Rad, USA).

Quantification of intact provirus was assessed using lysed extracts of CD4^+^ T cells. Duplex ddPCR was performed using the packaging signal (Ψ) and nonhypermutated envelope gene (*Env*) primer/probe sets, per the original Intact Provirus DNA Assay (IPDA) protocol using IPDA, as previously described [19]. The HIV packaging signal (Ψ) and the Rev responsive element in *Env* were amplified. For those samples that failed to detect the Env or packaging signal genomic region, we used a secondary prime/probe set to target an immediately nearby region of Env or U5’‐LTR, respectively, with the aim of rescuing provirus quantification [18, 20]. In parallel, two primer/probe sets targeting the *RPP30* gene were used to normalize cell counts and to correct for DNA shearing. Only individuals in whom both Ψ and Env could be amplified were included.

Viral transcription was evaluated by quantification of cell‐associated HIV‐RNA (caHIV‐RNA) in purified CD4^+^ T cells, using one‐step reverse‐transcription ddPCR [21]. The 5’‐LTR or *gag* genes and the housekeeping gene of TATA‐binding protein (*TBP)* were measured in parallel.

Finally, residual viremia (<50 HIV‐RNA copies/ml plasma) was measured using the ultrasensitive viral load (usVL) assay by ultracentrifugation of 9 ml of plasma with a limit of detection of 0.56 copies/ml, as previously described [22].

### HLA typing

High‐resolution HLA class I typing for B alleles was performed using sequence‐based typing methods. B*27 and B*57 were considered protective alleles, and B*35 and B*07 were considered risk alleles [9].

### *CCR5* genotyping

A portion of the *CCR5* gene was amplified by PCR with primers that flanked the 32‐bp deletion [23]. Wild‐type and deleted fragments of 185 bp and 153 bp, respectively, were generated and visualized in 2% agarose gels. Heterozygosity (*CCR5* wt/Δ32) was indicated by the presence of both fragments.

### Quantitative viral outgrowth assay

Leukapheresis was performed in 14 LoViReT individuals to quantify the size of the replication‐competent reservoir. A total of 38 million fresh CD4^+^ T cells per individual were used for a limiting dilution cell culture assay in the absence of autologous contemporaneous IgG [24]. Supernatants from day 14 were quantified with p24^Gag^ ELISA (PerkinElmer, USA). IUPM was determined using IUPMStats v.1 (https://silicianolab.johnshopkins.edu/) based on the maximum likelihood method. The detection limit was set at 0.0185 IUPM for all participants.

### Quantification of the HIV reservoir in anatomical compartments

Total HIV‐DNA was measured in anatomical compartments from secondary lymphoid tissues, including rectal biopsies and lymph node biopsies in LoViReT individuals with an IUPM lower than 0.1. In the case of rectal biopsies, 4–8 endoscopic specimens per individual were collected and processed immediately to minimize loss of lamina propria leukocytes (LPL) and/or bacterial contamination. The specimens were processed following the previously reported LPL‐vDNA protocol [18]. CD45^+^ cells from the LPLs were sorted in a BD FACSAria II flow cytometer using the anti‐CD45 (FITC) antibody. For comparison purposes, we used historical data from HIV‐infected individuals receiving ART, which were processed using the same protocol.

Lymph node biopsies were processed using a noninvasive scanner‐guided fine needle biopsy technique to aspirate cells from punctures of two inguinal nodes. Cells were placed in RPMI 1640 containing 1% penicillin/streptomycin and 10% fetal bovine serum (FBS). In order to increase the sensitivity of the technique, we concentrated the infected cell population by measuring the reservoirs in memory CD4^+^ T cells defined as CD3^+^CD4^+^CD45RA^–^. Cells were sorted in a BD FACSAria II flow cytometer using the antibodies CD3 (APC), CD8 (APC H7), and CD45RA (FITC). For comparison purposes, we used data on integrated HIV‐DNA from individuals receiving ART [25, 26], which should be similar to or smaller than total HIV‐DNA in people on ART.

In both cases, isolated cells were lysed, and total HIV‐DNA was quantified using ddPCR. Undetectable samples are expressed as the limit of detection, which varies between samples depending on cell input.

### Cell sorting of CD4^+^ subpopulations

CD4^+^ T‐cell subpopulations were isolated by cell sorting from 175–450 million peripheral blood mononuclear cells (PBMCs) obtained from 14 LoViReT individuals using leukapheresis. PBMCs were incubated with live/dead (APC‐Cy7), CD3 (BV510), and CD4 (AF700) monoclonal antibodies. T‐cell maturation was based on the expression of the surface markers CD45RA (APC), CCR7 (PE Dazzle), and CD27 (FITC) in order to define T_N_ (CD45RA^+^CCR7^+^CD27^+^), T_CM_ (CD45RA^–^CCR7^+^CD27^+^), T_TM_ (CD45RA^–^CCR7^–^CD27^+^), T_EM_ (CD45RA^–^CCR7^–^CD27^–^), and T_TD_ (CD45RA^+^CCR7^–^CD27^–^). Subpopulations were sorted in a BD FACSAria II flow cytometer, and cells were lysed to measure total HIV‐DNA using ddPCR.

### Statistical analysis

The clinical characteristics of the study population were presented as percentages for categorical variables and as median and interquartile range (IQR) for continuous variables. LoViReT individuals and controls were matched using a multivariate approach based on the random forest method. The proximity distance between individuals provided by the random forest method was used to select the control closest to each LoViReT [27]. Differences between LoViReTs and controls were assessed using the Mann–Whitney test in the case of continuous variables and the Fisher exact test in the case of categorical variables. The Spearman correlation coefficient was calculated to analyze correlations between study variables. The analyses were performed with GraphPad (v8.02).

## Results

### Participant characteristics

We selected 22 LoViReT individuals and 22 controls, all of whom had been receiving suppressive ART for more than 3 years. Their clinical characteristics are summarized in Table 1. No statistically significant differences were observed between the clinical characteristics of the groups owing to previous matching. Based on clinical health records, 14 (64%) and five (23%) LoViReT individuals were classified as having initiated ART during the chronic phase or the early phase of HIV infection (i.e., within 6 months from the estimated acquisition of HIV). The date of acquisition of HIV could not be estimated in three participants because the data were missing.

**Table 1:** Clinical characteristics of the subjects included in the study

	LoViReT group (*n* = 22)	Control group (*n* = 22)	*p*‐Value
Age at HIV diagnosis (years), median (IQR)^a^	34 (28–39)	33 (30–37)	0.7
Females, *n* (%)^b^	5 (23)	2 (9)	0.22
* CCR5 *‐tropic virus, *n* (%)^b^, ^c^	12 (92)	18 (78)	0.56
HIV subtype B, *n* (%)^b^, ^c^	12 (92)	20 (95)	0.72
Mode of HIV acquisition MSM, *n* (%)^b^	10 (45)	12 (54)	0.52
Time since HIV diagnosis (years), median (IQR)^a^	17 (10–23)	20 (12–27)	0.52
Time receiving ART (years), median (IQR)^a^	13 (9–18)	17 (11–25)	0.12
Time with suppressed pVL (years), median (IQR)^a^	12 (9–16)	13 (11–16)	0.34
Time from ART to supressed pVL (years), median (IQR)^a^	0.25 (0.1–0.3)	0.4 (0.2–0.6)	0.26
Zenith pVL (log_10_ copies/ml plasma), median (IQR)^a^	4.0 (3.5–4.9)	4.6 (3.6–5.1)	0.46
CD4 T‐cell count			
Nadir (cells/μl), median (IQR)^a^	300 (235–439)	297 (219–420)	0.58
CD4 T‐cell count (cells/μl) at sampling, median (IQR)^a^	859 (527–984)	787 (573–1025)	0.86
Antiretroviral treatment			
Number of different regimens, median (IQR)^a^	5.5 (4.7–7.3)	6 (3.8–9.3)	0.55
Number of different families, median (IQR)^a^	3 (3–4)	3.5 (2–4)	0.59
Nucleoside reverse transcriptase inhibitors, *n* (%)^b^	22 (100)	22 (100)	>0.99
Non‐nucleoside reverse transcriptase inhibitors, *n* (%)^b^	16 (73)	20 (90)	0.24
Protease inhibitors, *n* (%)^b^	18 (82)	17 (77)	>0.99
Integrase inhibitors, *n* (%)^b^	13 (59)	12 (55)	>0.99
Entry inhibitors, *n* (%)^b^	3 (14)	0 (0)	0.23
Treatment initiation with non‐HAART, *n* (%)^b^	4 (18)	8 (36)	0.31

Abbreviations: ART, antiretroviral therapy; HAART, highly active antiretroviral therapy; IQR, interquartile range; LoViReT, Low Viral Reservoir Treated; MSM, men who have sex with men; pVL, plasma viral load.

^a^ *p*‐Value between groups: Mann–Whitney test.

^b^ *p*‐Value between groups: Chi‐square test.

^c^ Data from individuals in whom amplification of the *Env* gene was achieved.

### The LoViReT phenotype is independent of the HLA and *CCR5* genotypes

We determined whether host protective genetic factors were enriched in LoViReT individuals. To do so, we first measured the HLA class I alleles (Fig. S2a). Six LoViReTs and two controls had protective alleles (two HLA‐B*57 and four HLA‐B*27 vs. one HLA‐B*57 and one HLA‐B*27), though the differences were not statistically significant (*p* = 0.27). On the other hand, we did observe a tendency toward a lower frequency of risk alleles in the LoViReT group (one HLA‐B*07 and two B*35 in LoViReT vs. four HLA‐B*07 and six B*35 in controls), though again, the differences were not statistically significant (*p* = 0.07).

We also evaluated the frequencies of the *CCR5*Δ32 mutation in the cohort (Fig. S2b) and found that 27.3% of LoViReT individuals had a *CCR5*wt/Δ32 genotype compared with 21.4% of the controls (*p* > 0.99).

Our results indicate that neither protective nor risk host genetic factors are enriched in the LoViReT cohort.

### HIV‐DNA expression and HIV‐RNA expression are reduced in LoViReT individuals

**Fig. 1:**
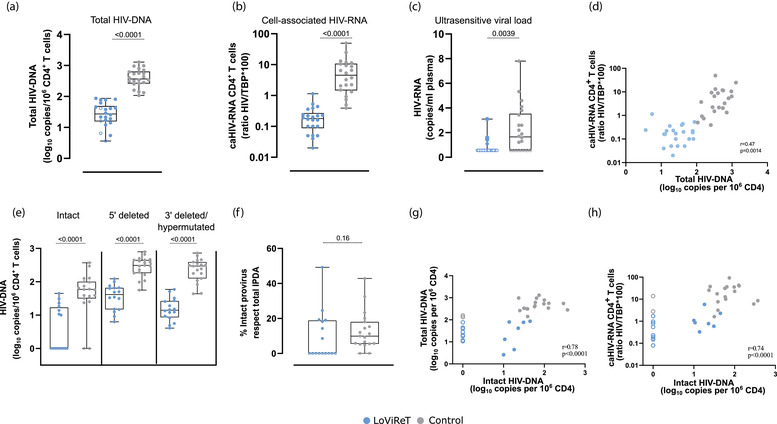
Measurements of total HIV‐DNA, HIV expression, and intact provirus. (a) Levels of total HIV‐DNA, (b) cell‐associated HIV‐RNA, and (c) ultrasensitive viral load in plasma. (d) Correlation between total HIV‐DNA and cell‐associated HIV‐RNA. (e) Levels of intact and defective provirus per 10^6^ total CD4^+^ T cells measured using Intact Provirus DNA Assay (IPDA). (f) Percentage of intact provirus with respect to total values using the IPDA. Total proviruses were determined as the sum of intact, 5′ defective and hypermutated/3′ defective proviruses from each individual. (g) Correlation between intact proviruses and total HIV‐DNA, and (h) correlation between intact provirus and cell‐associated HIV‐RNA. Open symbols represent values under the limit of detection; in these cases, the limit of detection varied based on sample/volume input. The Spearman correlation coefficient was calculated to analyze correlations between study variables.

As expected from the selection criteria, we observed statistically significant differences between the groups for levels of total HIV‐DNA measured in CD4^+^ T cells (*p* < 0.0001) (Fig. 1a).

In order to determine whether the low levels of total HIV‐DNA were also related to reduced residual viral replication, we analyzed levels of intracellular and extracellular forms of HIV‐RNA by measuring caHIV‐RNA and usVL in CD4^+^ T cells and plasma, respectively. HIV‐RNA expression was detected in all the LoViReT individuals, though levels were significantly lower than in controls (*p* < 0.0001) (Fig. 1b). Moreover, residual plasma viremia was detectable in only 19% of the LoViReT individuals, in contrast to 64% of controls (*p* = 0.0039) (Fig. 1c). In addition, significant positive correlations were found between total HIV‐DNA and caHIV‐RNA (r = 0.47, *p* = 0.0014) (Fig. 1d).

Therefore, the LoViReT phenotype is determined not only by low HIV‐DNA levels, but also by reduced expression of intracellular and extracellular HIV‐RNA in the context of viral suppression.

### Low levels of intact proviruses are also associated with LoViReT phenotype

To further analyze proviruses, we measured levels of intact provirus using IPDA. We observed lower levels of intact (*p* < 0.0001), 5′deleted (*p* < 0.0001), and 3′deleted/hypermutated (*p* < 0.0001) proviruses in LoViReT individuals (Fig. 1e). However, the frequency of intact proviruses with respect to the sum of intact, hypermutated/3'defective, and 5'defective proviruses did not differ between the groups (*p* = 0.16) (Fig. 1f). Significant positive correlations were observed between intact provirus and total HIV‐DNA (Fig. 1g) and caHIV‐RNA (Fig. 1h). Therefore, our results indicate that LoViReT individuals harbor low levels of intact proviruses, which were proportional to low levels of total proviruses, suggesting that there is no over‐representation of defective proviruses that could explain their phenotype.

### A low rate of undetectable replication‐competent virus in peripheral blood was observed in LoViReT individuals

**Fig. 2:**
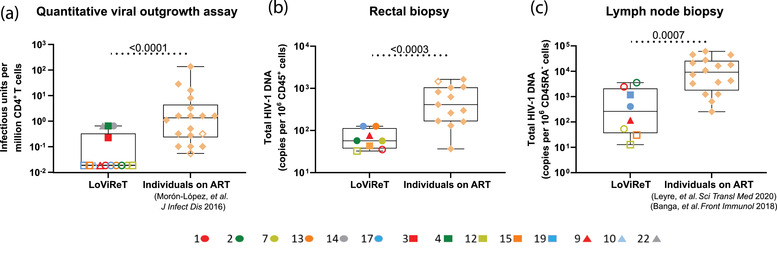
Measurements of the viral reservoir in peripheral blood and secondary lymphoid tissues. The figure shows data on each LoViReT (Low Viral Reservoir Treated) individual for (a) the viral outgrowth assay, (b) rectal biopsies, and (c) lymph node biopsies compared with HIV‐infected individuals receiving antiretroviral therapy (ART). LoViReT individuals treated in the chronic phase of infection are represented with a circle, those treated in the early phase of infection are represented with a square, and those with no available information on their status when they initiated ART are represented with a triangle. HIV‐DNA in rectal CD45^+^ and lymph node CD45RA^–^ was only assessed in LoViReT individuals with infectious units per million <0.1. The values from HIV‐infected individuals receiving ART are from Morón‐López et al. [28] for the viral outgrowth assay, and from Banga et al. [25] and Leyre et al. [26] for the lymph node biopsies. Median values are indicated by a horizontal black line. Open symbols represent values under the limit of detection; in these cases, the limit of detection varied based on cell input.

To determine whether the low levels of proviruses observed in LoViReT individuals were replication competent, we performed leukapheresis in a subgroup of 14 LoViReTs (Fig. 2a). We observed that 71% of the individuals had an IUPM under the limit of detection (0.0185), despite using up to 38 million CD4^+^ T cells to increase the sensitivity of the qVOA assay. The four LoViReT individuals with detectable qVOA had values ranging from 0.244 to 0.65 IUPM, which are all below the median IUPM of individuals on ART from previous studies [28].

### Low viral reservoirs were observed in secondary lymphoid tissues in LoViReT individuals

Since the latent reservoir may be unevenly represented in lymphoid tissues and peripheral blood, we explored potential secondary lymphoid tissue by collecting rectum and lymph node biopsies in a subset of eight LoViReT individuals with an IUPM <0.1 (Fig. 2a). Minimum levels of total HIV‐DNA were found in rectal samples, with detectable reservoirs in six of eight individuals (median of 56.9 HIV‐DNA copies/10^6^ CD45^+^ T cells [IQR: 37.4–113.7]) (Fig. 2b). These values were seven fold lower than historical data from our group on HIV‐infected individuals receiving ART (*p* = 0.0003).

Besides, only three out of eight individuals had detectable HIV‐DNA in lymph nodes, with a median of 262.6 HIV‐DNA copies/10^6^ CD45RA^–^ T cells (36.3–2112]) (Fig. 2c). In this case, the levels of HIV‐DNA in lymph nodes were statistically significantly lower for LoViReT individuals (*p* = 0.007) than for individuals receiving ART in previous studies [25, 26].

Matching of detectable/undetectable proviral HIV‐DNA reservoir in rectal biopsies agreed with peripheral blood findings in 75% of individuals. We also observed 57% agreement between rectal and lymph node biopsies.

In summary, LoViReT individuals have a lower or even undetectable reservoir compared with people on ART, not only in peripheral blood but also in specific lymphoid tissues such as rectum and lymph nodes.

### Low viral reservoir is independent of the time of initiation of treatment

**Fig. 3:**
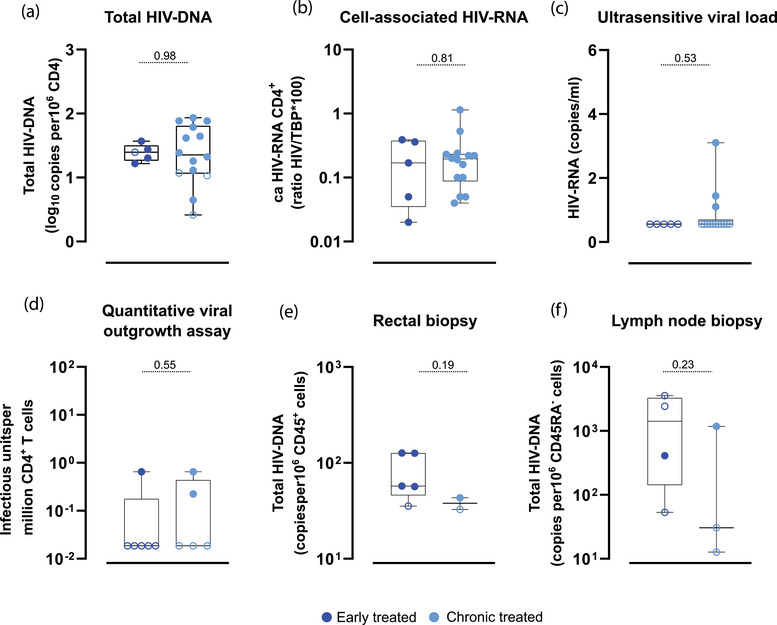
Measurements of total HIV‐DNA and HIV expression in LoViReT (Low Viral Reservoir Treated) individuals treated in the chronic or early phase of HIV infection. (a) Levels of total HIV‐DNA, (b) cell‐associated HIV‐RNA, (c) ultrasensitive viral load in plasma, (d) quantitative viral outgrowth assay, (e) HIV‐DNA in rectum biopsies, and (f) HIV‐DNA in lymph node biopsies. Open symbols represent values under the limit of detection; in these cases, the limit of detection varied based on sample/volume input.

Previous studies have shown a prospectively lower viral reservoir in individuals who started treatment early [29]; therefore, we questioned whether this effect was also observed retrospectively when we selected individuals with low‐level reservoirs during ART. We compared reservoir levels and HIV expression after classifying LoViReT into chronic‐treated LoViReT individuals (>6 months since HIV acquisition and initiation of ART, *n* = 14) and early‐treated LoViReTs (<6 months since HIV acquisition and initiation of ART, *n* = 5). We did not find statistically significant differences between the groups in peripheral blood reservoirs for total HIV‐DNA (*p* = 0.98), caHIV‐RNA (*p* = 0.81), usVL (*p* = 0.53), or replication‐competent proviruses (*p* = 0.55) (Fig. 3a–d). Similarly, with respect to lymphoid tissues, we did not find statistically significant differences between the groups in the median levels of total HIV‐DNA in rectum (*p* = 0.19) or lymph node specimens (*p* = 0.23) (Fig. 3e,f).

### Short‐lived CD4^+^ T‐cell subpopulations contributed more than expected to the HIV reservoir in LoViReT participants

**Fig. 4:**
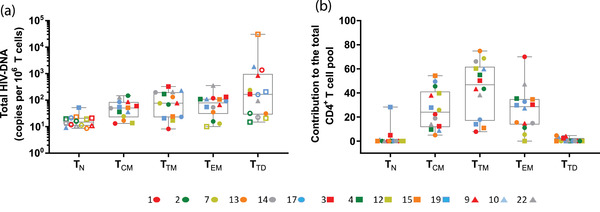
HIV latency distribution in T‐cell subsets in LoViReT (Low Viral Reservoir Treated) individuals. (a) Total HIV‐DNA measured using droplet digital polymerase chain reaction and (b) contribution of each subset to the HIV reservoir. LoViReT individuals treated in the chronic phase are represented by a circle, those treated in the early phase are represented by a square, and those with no available information on their status when they initiated antiretroviral therapy are represented by a triangle. Open symbols represent values that are below the limit of quantification; in these cases, the limit of detection varied based on cell input.

Finally, we aimed to determine whether the low level of proviruses observed in LoViReT individuals could be attributed to altered HIV persistence in a specific CD4^+^ T‐cell subpopulation. To do so, we sorted five CD4^+^ T‐cell subpopulations (T_N_, T_CM_, T_TM_, T_EM_, and T_TD_) from 14 LoViReT individuals and quantified total HIV‐DNA in each subpopulation (Fig. 4a). The reservoir was under the limit of detection in T_N_ and T_TD_ for most of the LoViReT participants (86% and 64%, respectively).

We then evaluated the contribution of each subset to the HIV reservoir by calculating the frequency of each subpopulation of CD4^+^ T cells (Fig. 4b). Both T_N_ and T_TD_ made negligible contribution to the HIV reservoir. However, unexpectedly, we observed that T_TM_ contributed the most to the total HIV reservoir (median of 47%), closely followed by T_EM_ and T_CM_ (median 29% and 24%, respectively).

Therefore, it appears that the distribution of the HIV reservoir in the CD4^+^ T‐cell subpopulations of LoViReT individuals is altered, thus potentially explaining the lower reservoir levels observed.

## Discussion

We previously described a new phenotype of HIV‐infected individuals on ART who harbor low levels of HIV‐DNA in peripheral blood, namely, the LoViReT cohort [13]. These individuals had reduced reservoir levels in PBMCs, even before initiation of ART, though their median viral loads were close to 10^5^ HIV‐RNA copies/ml, that is similar to those of control individuals [13]. However, the nature, distribution, and functionality of this small HIV reservoir remain unknown. Here, we studied 22 LoViReT participants to understand the mechanism behind their low HIV reservoir in blood and lymphoid tissue.

Our results confirm not only a low level of HIV reservoirs in LoViReT individuals in CD4^+^ T cells, but also that these proviruses were less transcriptionally active and produced fewer new virions. This finding took the form of lower detectability of residual plasma viremia. Our observations are in line with data from individuals with low levels of HIV‐DNA, such as elite controllers [2], exceptional elite controllers [4], and post‐treatment controllers [6], in whom significantly lower transcriptional HIV‐RNA activity and lower residual plasma viremia have also been observed.

In addition, when we measured the nature of the HIV reservoir in peripheral CD4^+^ T cells, we found lower levels of intact proviruses in LoViReT participants. Nevertheless, those differences seemed to be the consequence of the lower levels of total reservoir, since the proportion to total proviruses did not differ between the groups. In addition, we observed a positive correlation between total and intact proviruses. Interestingly, levels of intact proviruses were undetectable in nine individuals in the LoViReT group (56%) and two individuals in the control group (10.5%), the latter being consistent with the 12% reported elsewhere [20]. Thus, our IPDA technique is in line with the limit of detection validated in several laboratories.

To complement the molecular measurements, we set up a highly sensitive viral outgrowth assay based on ex vivo stimulation of 38 million peripheral CD4^+^ T cells. We observed that 71% of LoViReT individuals did not have replication‐competent proviruses (i.e., IUPMs <0.0185). Recent studies have shown that autologous contemporaneous IgG antibodies, if added to the culture, can block outgrowth of a substantial fraction of viruses in the latent reservoir for HIV‐1 by direct neutralization of the virus [30]. Therefore, the already low frequency of reservoir viruses capable of outgrowth in the absence of autologous IgG might represent an overestimation of the replication‐competent reservoir in these individuals. To our knowledge, this low level of replication‐competent virus has only been associated with extreme phenotypes of control, such as elite controllers or individuals who have undergone allogeneic stem cell transplantation and remain on successful ART [3, 4, 24].

Despite the low level of replication‐competent proviruses, recent studies have shown that analytical treatment interruption in individuals with characteristics similar to those of LoViReT predominately led to a viral rebound within 2–12 weeks after stopping ART [15, 16, 31]. For that reason, it is important to know whether lymphoid tissue plays a role in the rapid viral rebound observed when ART is interrupted. HIV reservoirs have been widely reported to be 5–12 times larger in lymphoid tissue, such as gut‐associated lymphoid tissue [32, 33] and lymph node [34], than in peripheral blood. Our results showed that LoViReT individuals have limited proviruses in the rectum and lymph nodes and that, in some cases, these even fall below the limit of detection. Median levels of proviruses in the rectum are only twofold larger than in peripheral blood. Moreover, when compared with historical data from studies performed in individuals on ART, we observed that reservoirs in rectal biopsies were 7.2‐fold higher than in the LoViReT individuals. Therefore, we concluded that the number of cells with HIV provirus in the rectum and lymph nodes is less predominant in this study group than in other individuals on ART.

The factor that might explain the total HIV‐DNA dynamic observed in these individuals is the distribution of the proviruses in CD4^+^ T‐cell subpopulations. Short‐lived T_TM_ and T_EM_ contributed considerably to the total HIV reservoir in LoViReTs. The rapid turnover rate of these cells [35] contributes to the expansion of identical genetically intact proviruses [36]. However, memory CD4^+^ T cells with a shorter cellular half‐life are also more easily cleared through successful ART [37], suggesting that they are not a durable reservoir in individuals receiving successful ART [38] and thus potentially explaining the more rapid decay of total HIV‐DNA in LoViReT individuals when they started ART [13].

Furthermore, T_TM_ and T_EM_ are characterized by higher levels of reactivation upon treatment with some latency‐reversing agents [39]; this could prove advantageous when trying new cure strategies aimed at reactivating the HIV reservoir. In contrast, in the general HIV‐infected population, the latent reservoir is found predominantly in the long‐lived cells such as T_CM_ [40], while in LoViReT individuals, long‐lived CD4^+^ T cells—including T_N_ and T_CM_—accounted for only 24% of the total HIV reservoir. This limited contribution of T_CM_ is also found in the following scenarios: the classic sooty‐mangabey model of attenuated SIV infection related to low *CCR5* coreceptor expression [41]; HIV‐2 infection, where the virus is less pathogenic and replicative [42]; post‐treatment controllers [6]; and long‐term nonprogressors associated with protective HLA‐B*27 or B*57 alleles [43].

Although LoViReT individuals have an interesting phenotype with respect to reduction of the HIV reservoir, this cohort differs from the previously described phenotypes of control. For example, 13% of post‐treatment controllers had been treated in the early phase of the infection while only 4% had been treated in the chronic phase [14]. Interestingly, we found that the time of initiation of treatment does not affect the levels of proviruses in LoViReTs, either in peripheral blood or in secondary lymphoid tissues. This observation contrasts with data from prospective studies, where the earlier ART is started, the smaller the reservoir becomes [44, 45]. LoViReT individuals, in particular, are characterized by a lower reservoir and better immune preservation before initiation of ART, which is followed by faster HIV‐DNA decay observed under ART [13]. Thus, the LoViReT phenotype appears to be defined by other factors that are independent of the time of initiation of treatment.

Control of HIV is characterized by the over‐representation of protective HLA‐class I alleles in long‐term nonprogressors [43], elite controllers [9, 10, 11], and exceptional elite controllers [4] and by risk alleles in post‐treatment controllers [6]. We found that protective alleles were slightly more frequent in LoViReTs, though the differences did not reach statistical significance. In contrast, other studies on individuals similar to those in the LoViReT cohort showed enrichment in HLA protective alleles, though this was not enough to control viremia after discontinuation of treatment [16]. In our cohort, it seems that other factors at the transcriptome or protein level might be involved in protecting T_CM_ from infection and that this in turn might have helped to attain some degree of control in establishment of the HIV reservoir. Nevertheless, it would be interesting to explore whether integration of the proviruses could be undertaken at sites that are not transcriptionally active, as recently shown for elite controllers [5].

Our study has some limitations. First, we did not obtain leukapheresis or secondary lymphoid tissues from control individuals, and therefore those values were compared to historical controls. In addition, given that we have a limited number of LoViReT individuals, we anticipate having a validation cohort, for which screening has already started, and that will contribute to deciphering the mechanisms of reduction of HIV reservoirs. Nevertheless, it is important to highlight that the screening of our current LoViReT cohort was carried out at two independent clinical sites in Barcelona, and a similar proportion of LoViReT individuals were obtained at both [13].

In conclusion, we showed that LoViReT individuals differ from other HIV control phenotypes. Factors associated with their status are related to impaired establishment of the reservoir in different lymphoid compartments and to altered proviral distribution towards CD4^+^ T‐cell subpopulations with shorter half‐lives. Their special characteristics might make them potential candidates for HIV cure strategies. These strategies should be aimed at further boosting the immune response in order to effectively control the potential residual virus that could reactivate from a minimal HIV reservoir in these individuals.

## Conflicts of interests

J. M.‐P. received an institutional grant and educational/consultancy fees from Merck Sharp & Dohme. Outside the submitted work, he has received institutional grants and educational/consultancy fees from AbiVax, AstraZeneca, Gilead Sciences, Grifols, Janssen, and ViiV Healthcare. The remaining authors declare that they have no competing interests.

## Author contributions

Cristina Gálvez, Javier Martinez‐Picado, and Maria Salgado conceived and designed the study; Cristina Gálvez, Maria del Carmen Garcia‐Guerrero, Sílvia Bernal, and Maria Salgado performed the experiments; Cristina Gálvez, Víctor Urrea, and Maria Salgado analyzed the data; Cristina Gálvez, Javier Martinez‐Picado, and Maria Salgado interpreted the results; Judith Dalmau, Susana Benet, Beatriz Mothe, Lucía Bailón, Andrea Martinez, Aroa Nieto, Bonaventura Clotet, Lorna Leal, and Felipe García contributed to the study design, recruitment of participants, sampling, and management of clinical data. Cristina Gálvez, Javier Martinez‐Picado, and Maria Salgado wrote the paper. All the authors read, reviewed, and approved the final version of the paper.

## Supporting information


**Supplementary Fig. S1** Study design flow‐chart. *Clinical factors used for matching of the groups included sex, age at diagnosis, age at sampling, CD4 T cells at sampling, CD8 T cells at sampling, % of CD4 T cells at sampling, % of CD8 T cells at sampling, CD4/CD8 ratio at sampling, number of blips, number of virological failures, zenith viral load, nadir CD4 T cells, time since undetectable viral load (50 HIV‐RNA copies/ml), total time with undetectable viral load, time from diagnosis to undetectable viral load, area under the curve for viral load normalized according to time undetectable, and AIDS events. IUPM = infectious units per million.
**Supplementary Fig. S2** HLA class I profile and *CCR5*wt/Δ32 frequency. (a) Frequency of the protective alleles HLA‐B*27 and B*57, risk alleles HLA‐B*07:02 and B*35, and genotype *CCR5*wt/Δ32 in LoViReT individuals and controls. (b) Frequency of individuals with the *CCR5*Δ32 mutation in heterozygosis.
