# Supplementary figures and images for: Altered T‐cell subset distribution in the viral reservoir in HIV‐1‐infected individuals with extremely low proviral DNA (LoViReTs)
Source: J Intern Med. 2022 Mar 28;292(2):308–20. doi: 10.1111/joim.13484 (PMC9308636; doi:10.1111/joim.13484)

Supplementary Figure 1

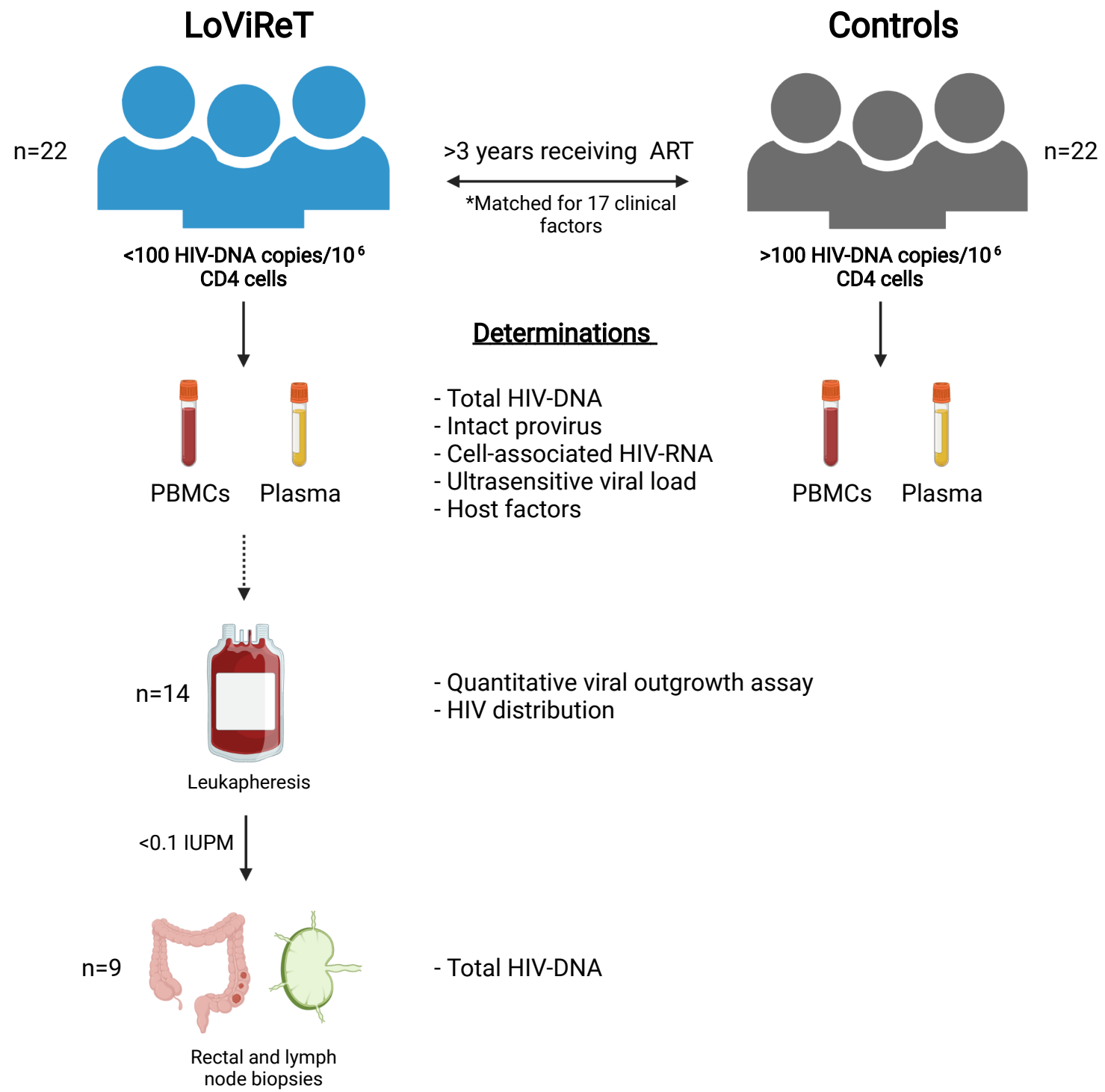

Supplementary Figure 2

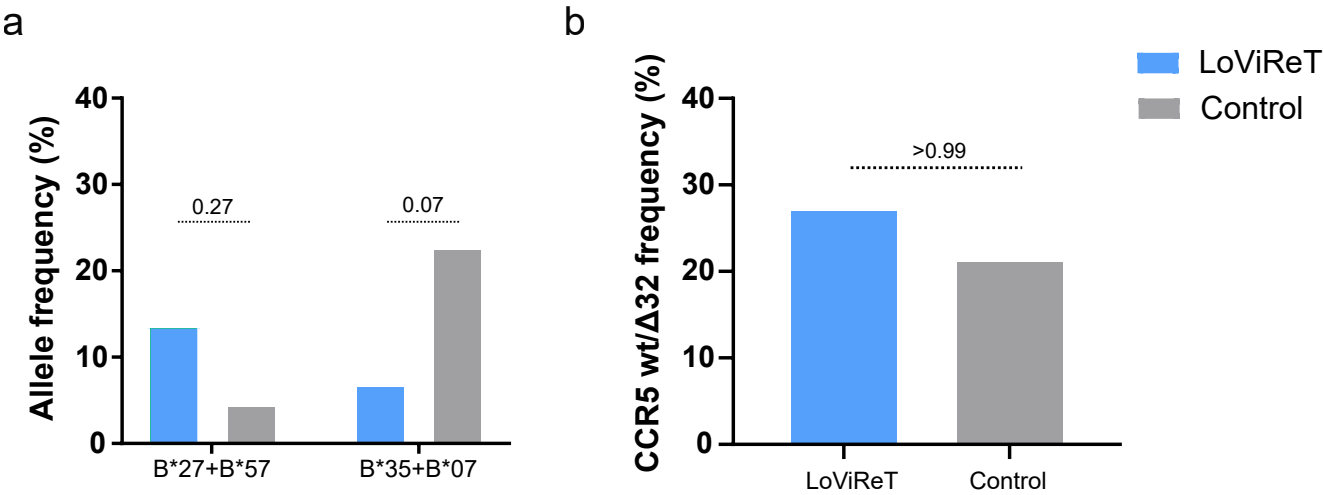

Supplement: Supplementary file 1 — Supplementary Fig. S1 Study design flow‐chart. *Clinical factors used for matching of the groups included sex, age at diagnosis, age at sampling, CD4 T cells at sampling, CD8 T cells at sampling, % of CD4 T cells at sampling, % of CD8 T cells at sampling, CD4/CD8 ratio at sampling, number of blips, number of virological failures, zenith viral load, nadir CD4 T cells, time since undetectable viral load (50 HIV‐RNA copies/ml), total time with undetectable viral load, time from diagnosis to undetectable viral load, area under the curve for viral load normalized according to time undetectable, and AIDS events. IUPM = infectious units per million. Supplementary Fig. S2 HLA class I profile and CCR5wt/Δ32 frequency. (a) Frequency of the protective alleles HLA‐B*27 and B*57, risk alleles HLA‐B*07:02 and B*35, and genotype CCR5wt/Δ32 in LoViReT individuals and controls. (b) Frequency of individuals with the CCR5Δ32 mutation in heterozygosis. [file JOIM-292-308-s001.pdf]
